# Securing Color Fidelity in 3D Architectural Heritage Scenarios

**DOI:** 10.3390/s17112437

**Published:** 2017-10-25

**Authors:** Marco Gaiani, Fabrizio Ivan Apollonio, Andrea Ballabeni, Fabio Remondino

**Affiliations:** 1 Department of Architecture, University of Bologna, Bologna 40136, Italy; fabrizio.apollonio@unibo.it (F.I.A.); andrea.ballabeni@unibo.it (A.B.); 2 3D Optical Metrology (3DOM) Unit, Bruno Kessler Foundation (FBK), Trento 38123, Italy; remondino@fbk.eu

**Keywords:** color acquisition, color balance, photogrammetry, automation, color management, structure for motion, photometric calibration

## Abstract

Ensuring color fidelity in image-based 3D modeling of heritage scenarios is nowadays still an open research matter. Image colors are important during the data processing as they affect algorithm outcomes, therefore their correct treatment, reduction and enhancement is fundamental. In this contribution, we present an automated solution developed to improve the radiometric quality of an image datasets and the performances of two main steps of the photogrammetric pipeline (camera orientation and dense image matching). The suggested solution aims to achieve a robust automatic color balance and exposure equalization, stability of the RGB-to-gray image conversion and faithful color appearance of a digitized artifact. The innovative aspects of the article are: complete automation, better color target detection, a MATLAB implementation of the ACR scripts created by Fraser and the use of a specific weighted polynomial regression. A series of tests are presented to demonstrate the efficiency of the developed methodology and to evaluate color accuracy (‘color characterization’).

## 1. Introduction

In the last years major progress has been achieved in the main areas of the automatic photogrammetric pipeline, i.e., scalable tie point extraction [1], large-scale bundle adjustment [2] and dense point clouds generation [3], i.e., the core components of the photogrammetric pipeline. These progresses allow today to reconstruct large scenes from image sequences readily and at low cost [4]. These achievements improve and speed-up the image-based 3D reconstruction pipeline, particularly in the Architectural Heritage (AH) field [5], but none of them provides the solution to the problem of accurate color reproduction using automated processes.

Nowadays, the problem of an accurate color description and reproduction using images could be depicted as the faithful determination of chromatic and tonal properties of the color [6]. These can be determined taking into account that the values of a color in an image are the result of the interaction of incident illumination, object geometry, object reflectance, lens and camera transfer function. The accuracy of the results of chromatic and tonal properties determination depends on several variables (i.e., illumination during the acquisition step, technical features of the imaging system, mathematical representation of color information throughout the acquisition and reproduction workflow, etc.). The best results are achieved with controlled light sources (i.e., artificial) in indoor environments, where the knowledge of the illumination allows to estimate surface reflectance parameters using image values [7]. AH, however, implies outdoor environments, where natural light features are complex and mutable.

Moreover, the wide range of materials in historical buildings and monuments façades leads to different values of light reflection, porosity, etc., notwithstanding the fact that the same material reacts to light in different ways, at different time and place. Therefore, designing a basis set of lighting conditions it’s impractical.

These difficulties increase when the problem of chromatic and tonal reproduction is addressed to the context of reality-based 3D modeling and visualization [8]. Furthermore, as automation in image processing increases continuously, high-quality image radiometry is becoming fundamental to allow automated tasks to perform correctly and to have successful and high-quality 3D photogrammetric reconstructions [9].

### Paper Contributions

**Figure 1:**
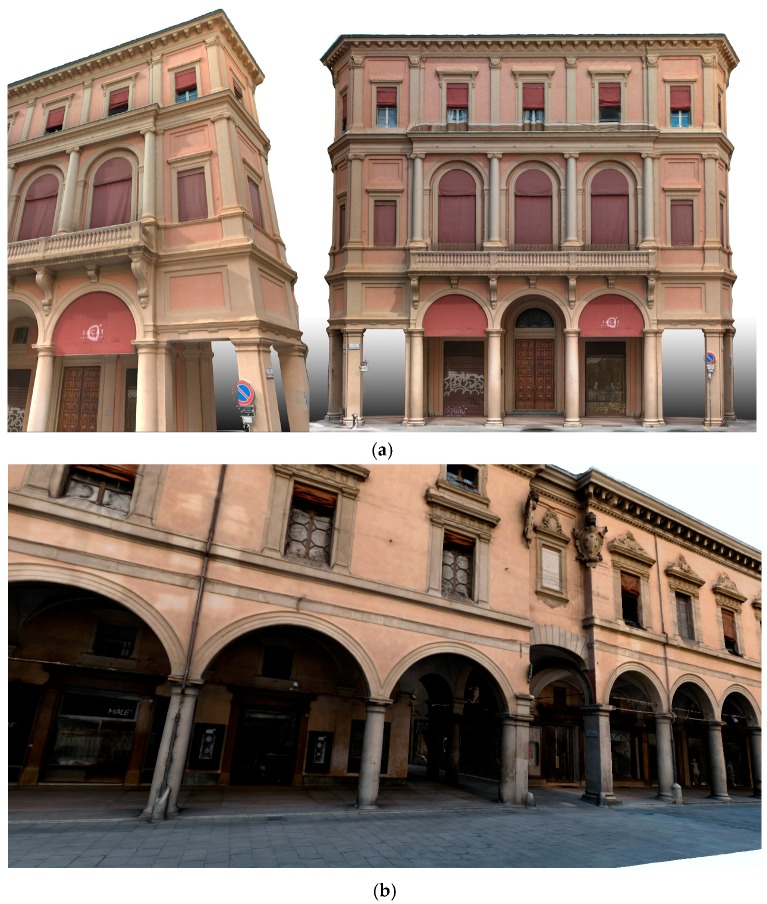

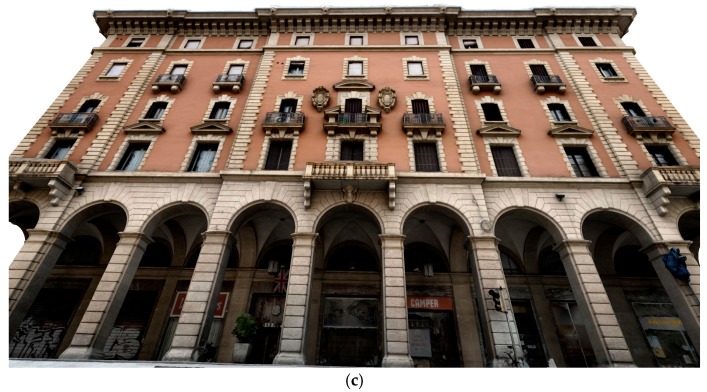
Results of the presented image-based 3D reconstruction of complex architectures (historical buildings with porticoes in Bologna) achieved with the presented methodology: (**a**) Palazzina in S.Tecla; (**b**) Palazzo dell’Archiginnasio; (**c**) Palazzo Assicurazioni Generali.

Given the abovementioned open issues, this paper presents an efficient and automated image pre-processing solution to accurately reproduce the correct colors and geometries of a surveyed AH (Figure 1).

This includes a radiometric improvement of the image quality and, at the same time, an increase in processing performances for the two central steps of the photogrammetric pipeline (i.e., image orientation and dense image matching). The methodology improves what presented in [10] where a process for the color acquisition and management during the generation of 3D AH models was proposed. Such process was successfully tested on the 3D digitization of 10 km of historical porticoes, but its applicability demonstrated some bottlenecks mainly due to the lack of automation in many steps.

The new methodology is based on simple and rapid procedures, standard and open source technologies, low-cost commercial instruments and it is completely automated in order to be integrated in daily workflows [11]. The pre-processing method could even be embedded in automated web-based applications (i.e., Autodesk ReCap, etc.) or offline desktop-based applications (i.e., Agisoft Photoscan, Pix4D Mapper, Capturing Reality RealityCapture, etc.).

The methodology consists basically in a pre-processing of RAW images used to define colors and shape. The output of the procedure is a set of color-calibrated images. RAW files are intentionally inscrutable data structures, but have been reverse engineered with some success to gain access to the raw data inside [12]. Automated image pre-processing of RAW images produces better photogrammetric accuracy if compared to JPEG images [13].

With the aim of avoiding as far as possible modifications of RAW pixel values, only the basic in-camera processing was considered: bad pixel removal, bias subtraction and flat-field correction, dark frame, black point subtraction, green channel equilibrium correction and Bayer interpolation. Denoising, color scaling, image sharpening, color space conversion, Gamma correction and format conversion were disabled in the camera and performed off-camera, including exposure compensation and color balance. This help to provide a linear relationship to scene radiance and to obtain device-independent images that can be quantitatively compared without knowledge of the original imaging system [14].

The proposed methodology (Section 4) aims to achieve a robust automatic color balance and exposure equalization to ensure (i) faithful color appearance of a digitized artifact and (ii) improvements in both sparse and dense 3D reconstruction in the photogrammetric process. The final goal is to enhance the photogrammetric results, ensuring better geometric accuracy and an improved fidelity of the color in the screen 3D visualization runtime. A software exploiting X-Rite ColorChecker Classic (CC) was thus developed and various tests were performed to demonstrate the efficiency of this procedure in several situations. Color accuracy (‘color characterization’) was evaluated and expressed. Finally, the color enhancement pipeline was compared within the image-based reconstruction pipeline in three contexts: matching of image correspondences, image orientation and dense image matching.

## 2. Camera Radiometric Calibration and Color Space Transformation

A digital image captured with a digital camera is formed by the intensity values of the RGB channels. Each of these values is influenced by three physical characteristics: (i) source of light (also called illuminant); (ii) object reflectance or transmittance and (iii) sensor spectral response (combination of spectral characteristics of colorants used in the color mosaic filters and spectral sensitivity of the photodetectors). As stated in [15,16] the image values *f_c_* = (*f_R_*, *f_G_*, *f_B_*) depend on the color of the light source *I*(*λ*), the surface reflectance *S*(*x*,*λ*)and the camera sensitivity function *ρ*_C_(*λ*) = (*ρR* (*λ*), *ρG* (*λ*), *ρB* (*λ*)), where *λ* is the wavelength of the light and *x* is the spatial coordinate:

(1) $$f_{c}\left(x\right)=m_{b}\left(x\right)\int_{\omega }I\left(\lambda \right)\rho_{c}\left(\lambda \right)S\left(x,\lambda \right)d\lambda +m_{s}\left(x\right)\int_{\omega }I\left(\lambda \right)\rho_{c}d\lambda$$

*c* = *{R,G,B}*, *ω* represents the visible spectrum, and *m_b_* and *m_s_* mean scale factors that model the relative amount of body and specular reflectance that contribute to the overall light reflected at location *x*. According to the Lambertian assumption the specular reflection is ignored. This results in the following model:

(2) $$f_{c}\left(x\right)=m\left(x\right)\int_{\omega }I\left(\lambda \right)\rho_{c}\left(\lambda \right)S\left(x,\lambda \right)d\lambda$$

where *m*(*x*) represents Lambertian shading. We assumed that the scene is illuminated by one single light source and that the observed color *e* depends on the color of the light source *I*(*λ*) as well as on the camera sensitivity function *ρ*(*λ*):

(3) $$e=\left(\begin{array}{c} \begin{array}{c} e_{R}\\ e_{G} \end{array}\\ e_{B} \end{array}\right)=\int_{\omega }I\left(\lambda \right)\rho \left(\lambda \right)d\lambda$$

both *I*(*λ*) and *ρ*(*λ*), without any prior knowledge, are unknown and hence the estimation of *e* is an ill-posed problem that cannot be solved without further assumptions.

To estimate the sensitivity functions of the camera, many computational camera calibration models were developed [17,18]. Basically, the problem of color correction for digital images stems from the impossibility to represent camera sensor sensitivities as a linear combination of CIE color matching functions, since they violate the Luther-Ives conditions [19]. Surfaces dissimilar to the eye could bring the same camera responses and vice-versa, in a camera-eye metamerism [20]. Color correction is unable to solve this metamerism, but it allows to establish the best possible mapping from camera *RGB*s values to device independent *XYZ*s values (or display sRGBs values [21]). ‘Color characterization’ refers to techniques allowing to convert camera responses (i.e., *RGB*) to a device-independent colorimetric representation (i.e., CIE*XYZ*) [22].

The main problem faced by ‘color characterization’ techniques consists in finding a linear relationship between the irradiance values and the typically non-linear camera pixel encoding introduced by in-camera processing to enhance the visual quality of captured images. The ‘color characterization’ problem is usually solved shaping the non-linear camera response function from observations. Different variants were developed to do this: multiple images taken with different exposure times from a fixed camera location [23,24], color distribution analyses nearby image edges [25], different color profiles from different lightings [26]. Using these techniques, radiometric response functions recovering is an ill-posed problem without additional knowledge or assumptions.

Basically, ‘color characterization’ methods are divided into two general groups in the literature: spectral sensitivity-based and color target-based approaches, as specified by ISO17321.14 [27].

Spectral sensitivity-based methods connect device-dependent and device-independent color spaces by a linear combination of camera spectral sensitivity curves and color matching functions [28,29,30]. As described in [9], the response of the camera to each wavelength can be measured using a specialized instrument such as a monochromator and a radiance meter. Then the relationship between responses and CIE device-independent colorimetric representation can be estimated.

The target-based characterization methods establish the color relationship according to a set of color patches with available pre-measured spectral or colorimetric data [31]. These techniques use a set of differently colored samples that can be measured with a spectrophotometer. The material aspects become important particularly when color patches vary in surface and spectral properties, leading to different appearance changes according to geometry changes. Target-based characterization methods are valid for a lighting geometry, color target materials, and a specific surface structure. This dependence led to designing optimized color targets in recent research, considering several factors, such as material, gamut, and the number of color patches. The variety of capturing geometries and the uncontrolled nature thereof are other factors increasing the complexity of the characterization process.

As reported in [32], the most accurate target-based characterization requires to record its output for all possible lighting and exposure and comparing it with separately measured values for the same lighting and exposure conditions. However, this process generates a massive and unmanageable amount of data. Therefore, the device response is measured only for a selected set of stimuli (e.g., for different CIE illuminant should be done different characterizations, but typically two D series illuminant (daylight) behave in the same way). Starting from this limited set of responses, the transformation between measured CIE*XYZ* values and captured *RGB* values is recovered using one of the techniques among linear transformations, multidimensional lookup tables [33,34,35], least-squares polynomial regressions [36], advanced regressions [37], neural networks [38] and human-observation-based models [39]. A complete review of these techniques is given in [7].

In many cases, a simple linear transformation is sufficient to map device-dependent and device-independent spaces with adequate performance. A linear transformation, named colorimetric matrix, gives an estimated tristimulus value $\left(\hat{X_{i}},\hat{Y_{i}},\hat{Z_{i}}\right)$ from $\left(R_{i,}G_{i,}B_{i,}\right)$, i.e., assumes that the camera spectral sensitivities are linear combinations of the device-independent ones. The colorimetric matrix is usually obtained through least-squares minimization [40]. An efficient solution to compute this matrix is to minimize the total visual color difference *M*, which is a weighted sum of the color differences ∆*E* (computed e.g., in the CIE perceptual uniform color space Lab) between the target tristimulus $\left(X_{i,}Y_{i,}Z_{i,}\right)$ and its estimate $\left(\hat{X_{i}},\hat{Y_{i}},\hat{Z_{i}}\right)$, for each patch *i*, 1 ≤ *i* ≤ *n*:

(4) $$M=\overset{n}{\underset{i=1}{\sum }}w_{i}\Delta E\left(X_{i,}Y_{i,}Z_{i,}\hat{X_{i}},\hat{Y_{i}},\hat{Z_{i}}\right)$$

where *w_i_* are the weights for the different patches. 

Usually the color error parameter is the mean of Δ*E*_00_, which is calculated for all patches where 5 ≤ L* ≤ 98 (where L* is defined in CIELAB color space) and each of the R, G, and B channels are less than 99% of the maximum value (i.e., the patch is unsaturated) [41].

Linear approaches are extensively employed for ‘color characterization’ as they preserve two key properties directly related to the camera sensors linear response to the light sources: scalability and hue planes [42]. The scalability property means that the characterization is invariant to camera exposure changes (i.e., the same surface viewed under different light levels in different parts of the scene). Therefore, a linear map is the same for the different exposure values. This is not true for most non-linear characterizations, where a change in exposure will scale *RGB* values, but could generate angular shifts in the *XYZ* vectors, producing visible color shifts.

Moreover, linear transformation well performs in most digital cameras. Generally, camera systems have nearly equal response curves for all channels, and it is possible to use a single mapping for all three channels. However, it can happen that the camera’s response is different per each channel. To handling this, the two most usual approaches consist in the use of a weighted average of the channels, and a different ‘color characterization’ for each channel. This last technique presents the problem that the white point is not preserved (i.e., the white in *RGB* is not correctly mapped to white in the CIE*XYZ* color space [43]). The method is then accurate only under certain standard illuminants because the characteristics of the color imagery system depend on the surrounding illumination. An additional term can be added to prevent this [44] and more accurate and robust techniques were proposed [45].

Despite above listed benefits, linear correction may produce significant errors. To allow better estimations an efficient option is the polynomial color correction obtained extending linear correction with additional polynomials of increasing degree [46]. The polynomial regression method based on least squares fitting has been widely adopted in colorimetric characterization because of its simplicity and good performance [47,48]. For fixed calibration settings, a polynomial regression can strongly reduce the mapping error [37] allowing significant improvements to color correction. However, the use of high degree data expansions can result in unstable (rank deficient) data sets as demonstrated in [49]. This problem can be mitigated by regularizing the regression [50].

Another major problem in the use of the polynomial regression consists in its not scale-independent feature (i.e., data scaled as result of changes in the scene radiance or exposure). If data are scaled, then the best color correction matrix must also change. This shift can be significant in several cases and this is a significant problem in outdoor captured images. Analogously, the planes of constant hue (i.e., the planes of a matte surface of constant hue parts in light and some in shadow) will be preserved in a linear transformation, but not with a non-linear transform, where could appear color shifts. Then, for polynomial regression, the colorimetric matrix needs to be recalculated for each exposure change. Recently some solutions have been proposed for retaining scalability [51,52]. In the ‘Root-Polynomial’ regression [53] the nth root of each nth order term in the extended polynomial vector is taken. Tests show that for fixed illumination, root-polynomial based ‘color characterization’ perform comparably to standard polynomial correction. And a small change in the scene radiance or exposure produces significantly better results. In [54] it is demonstrated that even though ‘Root Polynomial’ color correction allows to minimize the effect of camera exposure and illumination changing, the technique is sensitive to gradients of scene irradiance. To overcome this drawback, [54] normalizes the *RGB* and *XYZ* vectors prior to performing root-polynomial regression, obtaining a scheme improving max Δ*E*_00_ but not the mean.

A last general observation concerning all developed algorithms (linear and non-linear) for target-based ‘color characterization’, pertains the need to consider noise for color correction, as they amplify the noise of the image. To limit this drawback, Lim & Silverstein proposed spatially varying color correction (SVCC), dividing the image into local blocks and estimating the optimal color correction matrices for each local block considering the noise effect [55]. While these algorithms can successively suppress the noise amplification, the noise is still included in the color corrected image because these algorithms do not explicitly incorporate denoising functionality [56]. To overcome this problem, Takahashi et al. [56] propose a color correction consisting of two parts: (a) application of the SVCC to correct the color of the noisy *RGB* image while suppressing the noise amplification; (b) application of a denoising framework—based on BM3D algorithm [57] and an estimation of noise level of input image—to the color corrected image with spatially varying noise levels after the SVCC. The developed solution presented in this paper is inspired by this method and tries to limit its complexity.

## 3. Target Detection

In the camera characterization techniques based on color targets, the targets placed in the scene are usually manually or semi-automatically localized to extract color values of chart patches.

**Figure 2:**
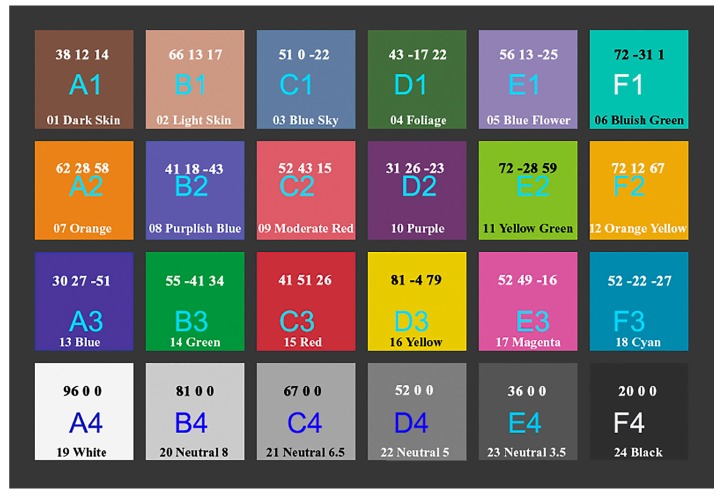
X-Rite Color Checker (CC) Classic array, with CIELab values.

The most common solution for target-based characterization techniques is the target Color Checker (CC) [58], which has standardized patches with known reflectance. It is a matte chart with dimensions 279.4 mm × 215.9 mm consisting of 24 patches, applied to paper then mounted to a cardboard backing with a black frame around all the patches. Each patch is 40 mm of side sized and the patches are organized in a 4 by 6 array, with 18 familiar colors that include the representation of true colors of natural matter (such as skin, foliage and sky), additive and subtractive primary colors, six grayscale levels with optical densities from 0.05 to 1.50 and a range of 4.8 f-stops (EV) (Figure 2).

When dealing with large image datasets the target localization can be lengthy and tedious. Automatic target detection is then an important feature and become a needed requirement in automated processing pipelines. Currently only few proposals exist addressing automatic color target detection. In [59] a semi-automatic method focusing on images with a significant degree of distortion is introduced. The presented technique requires an operator to select the four corners of the chart. On this basis, the coordinates of color patches are estimated using projective geometry.

The essence of this method is a multi-stage color patch detection, where the image is at first transformed with a Sobel kernel, then edited by a morphological operator to foster thresholding into a binary image, ready for final finding of connected regions. This technique is not suitable in this study, since it requires an operator.

The authors of [60] introduce a real-time automatic color target detection procedure extracting polygonal image regions and applying a cost function to check adaptation to a color chart. The color chart model is described by coordinates and color values of chart patches. To find the chart coordinates in image, four corners of chart model are projected on image with the use of a Direct Linear Transformation (DLT). Coordinates of color chart are found and a cost function measures the colors of the chart in the image and determines correction parameters. The technique is fast and robust against image noise, blur and other typical distortions, but image region statistics fails when color target is very small in the imaged scene.

In [61] image is binarized at first, patches are grouped and bounding parallelograms identified. Heuristics are then used to identify the color chart type.

In [62] target detection is performed using a different approach, in which detection relies on illumination changes between background and foreground. These authors use a coarse-to-fine methodology to localize the chart and recover its shape, i.e., the position and the edges of each color patch. Target detection is achieved through a two-step process: first of all, image features are roughly extracted and matching a sliding rectangle to a template color checker reproduction; after the color chart is approximately localized, a more precise detection and localization is accomplished.

A different class of solution aims to simply locate the color target in the image rather than detect it. In [63] authors present an automatic CC detection technique where the algorithm firstly quantizes all measured hues to the color chart ones; after that, the algorithm uses component analysis to find patch candidates; a Delaunay triangulation is then computed to locate the definitive candidate patches to be able to establish the chart position and orientation.

These methods assume low perspective distortions, a known scanning resolution and an approximately constant lightning in the image.

A more general approach is proposed by [64], who used color descriptors to automatically locate the color reference target. SIFT feature matching and then clusters matched features are fed into a pose selection and appearance validation algorithm. The method is robust to varying illumination conditions, but it is complex and presents the limits of SIFT technique.

Commercial software is capable of doing a semi-automatic color reference target detection. Examples are the X-Rite ColorChecker Passport Camera Calibration Software [65], Imatest [66] and BabelColor PatchTool [67]. Each software, however, usually relies on human intervention to manually mark or correct the detected reference target.

Freely available tools also exist. The most suitable applications are MacDuff [68] and CCFind [69], both aiming at detecting the CC inside an image. MacDuff, exploiting some code from OpenCV, handles the case of images with an X-Rite ColorChecker Passport. The solution uses adaptive thresholding followed by contour finding with heuristics in order to filter down to ColorChecker squares, then using k-means clustering to cluster squares. The average values of colors of square patches are then computed and a recursive process based on Euclidean distance in *RGB* space minimization try to find if any layout/orientation of square clusters would match CC reference values. The software assumes that the CC occupies a relatively large portion of the input image.

CCFind is a method implemented in MATLAB specifically designed not to use color as a cue. It does not detect squares explicitly, but it recognizes and understands the recurring shapes inside an image. It’s a shape-based solution robust to illumination changes and perspective distortion, returning the coordinates of the center points of the color patches of a CC. The main limitation is the difficulty to find the CC, relatively small within the image or near the image borders.

García Capel and Hardeberg implemented a preprocessing step for finding an approximate region of interest (ROI) of a CC, and apply it to both CCFind and a template matching approach [70]. The algorithm uses a texture transformation to better differentiate the reference target region from the rest of the image (i.e., to enhance its contrast). Then it creates a saliency map by using region and color information, and a threshold is used to create a binary mask of this saliency map. A ROI is finally determined by applying morphological operations to the resultant mask. The experiments show that the algorithm improves both detection and computation time by reducing the search area, but further improvements are needed to use it in a fully automated workflow.

This review shows that main difficulties in color target detection are basically: deformation and distortion of shape of CC; small size of CC in the image; light conditions. In detail, the shape deformation of target affects the finding of the correct orientation and location of the chart in the image. Distortions arises from the location of color chart, not rigid material of color chart and camera optics. Ambient light originates fluctuations in the color values due to non-uniformity. Given these considerations and the typical scenarios of our work, a new approach was developed.

## 4. Developed Techniques

The aim of the developed color pre-processing consists essentially in obtaining radiometric calibrated images able to ensure the consistency and fidelity of surfaces colors reproduction throughout the photogrammetric pipeline. This brings not only a benefit to the texture-mapping phase, but it also improves both sparse and dense reconstruction in the photogrammetric pipeline.

The problem of ‘color characterization’ is solved using a target-based technique exploiting the popular and consistent CC. Reference values were measured with a spectrophotometer. The developed technique is a two steps procedure: firstly, a polynomial regression technique is used; then an optimization algorithm is applied with the aim of refining the results and overcoming the limitations of the polynomial regression technique. This ‘color characterization’ technique is combined with the CIE color metric (ΔE*_00_) [71]:

(5) $$\Delta E_{00}=\sqrt{{\left(\frac{\Delta L^{\prime }}{k_{L}S_{L}}\right)}^{2}+{\left(\frac{\Delta C^{\prime }}{k_{C}S_{C}}\right)}^{2}+{\left(\frac{\Delta H^{\prime }}{k_{H}S_{H}}\right)}^{2}+R_{\tau }\frac{\Delta C^{\prime }}{k_{C}S_{C}}\frac{\Delta H^{\prime }}{k_{H}S_{H}}}$$

which is given by the CIE in 2000. It takes into account the problem of non-perceptual uniformity of the colors for which ΔE*_00_ varies the weight of L* depending on where the brightness range falls. It allows more accurate results in our cases. The formula, even if it shows some discontinuities, is more accurate than other color error metrics (e.g., Δ*E*_94_) and it is recommended by CIE mainly for color differences within the range 0–5 CIELAB units [72,73]. 8-bit sRGB by Denny Pascale [74] have been used as reference for CC built before November 2014 and measurements with a spectrophotometer for CC built after November 2014. To reduce the impact of noise on the color correction, a noise reduction strategy has been introduced. The ‘color characterization’ technique minimizes the CIE ΔE*_00_ differences between the standard and theoretical *XYZ* values to calculate the calibration matrix and then it applies an optimization iterative process based on six color patches (row 3, RGB and CMY), and the patch D4 of the CC (Figure 2).

The typical application scenario is the calibration process used with groups of images (50–100 images) having the same features (orientation, exposure, surfaces framed), a situation that is met when all the shots of a specific scene are taken in very short time (few minutes) and with similar point of view and camera direction, and then illumination and camera parameters remain practically unchanged. A captured color image containing the CC is neutralized, balanced and properly exposed for the gamma of the reference data set. Then found parameters are applied to the whole images group. Thus, a building can be modeled by less than 4–5 different ‘color characterization’ parameters, thereby maintaining consistency in process and results.

Basically, the developed software solution is a RAW image processing implemented in MATLAB and supported by RawTherapee, a cross-platform open source raw image processing program, written in C++, using a GTK+ front-end and a patched version of DCRaw for reading raw files [75]. It allows advanced control over the demosaicing, devignetting, white balance output file in a rendered color space, gamma correction, and 8-bit/16-bit conversion without the need of further software development, exploiting command-line interface easily coupled with MATLAB [76].

**Figure 3:**
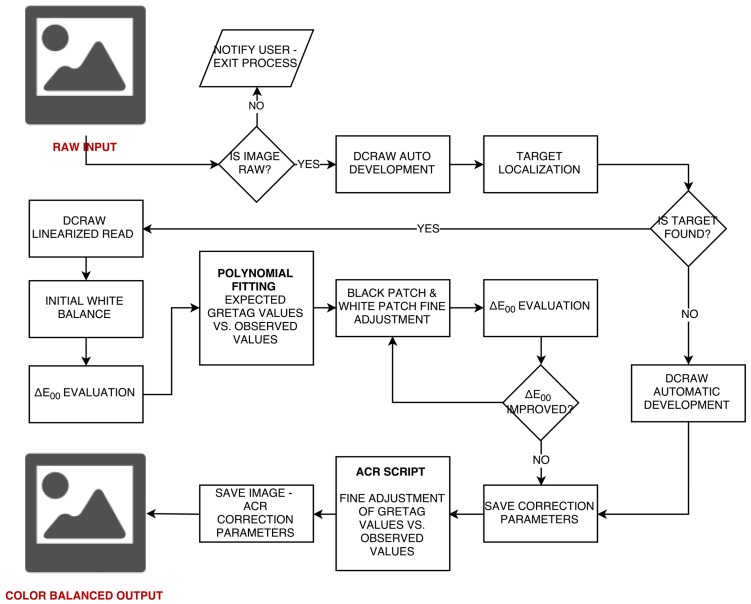
Radiometric calibration workflow presented in the article.

The workflow, illustrated in Figure 3, is as follows:

1. RAW image 16-bit linearization and devignetting.
2. Image denoise.
3. ColorChecker (CC) detection.
4. Exposure equalization and white balance exploiting the patch D4 of the CC.
5. Polynomial fitting of observed values with expected values.
6. Image correction using the fitting function found at point 3.
7. Color balance of the correct image.
8. ΔE*_00_ mean error evaluation on the CC.
9. Iterative image correction using the fitting function found at point 3 increasing the degree of the polynomial at each step; iteration stops when ΔE*_00_ stops decreasing.
10. Image correction using the new fitting function.
11. ACR-like scripts calibration.

**Figure 4:**
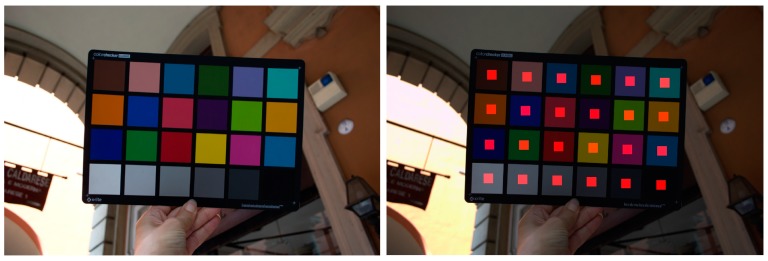
Result of CC detection.

Color space assignment in the sRGB color space. Main features of the developed software are as follows:

(a) *ColorChecker detection*. The solution presented in [70] has been improved correcting the failures when the target was too near to the observer, basically due to wrong exposures, non-uniform lighting of the CC, or CC warped in the image. The algorithm, if it does not find the target at first attempt, resizes the image increasingly and adds a frame of white pixels around it, to keep the initial size. After the target is found in the image resized, coordinates are brought back to the original ones. A further step was added to avoid residual failures switching to the original solution [69] allowing to enable the search of the CC outside of the found approximate region of interest (ROI) (Figure 4).
(b) *RAW image linearization*. It is possible that the camera applied a non-linear transformation to the sensor data for storage purposes (e.g., Nikon cameras). However, to calculate the color correction that can be applied to images in order to achieve optimum color reproduction, which is defined as the colors that have minimum color error on the test chart of choice, it is very important that the image is properly linearized because the corrections algorithm operates on linear R, G, B values and the RAW file is not usually a linear image. The image was then linearized to CIE*XYZ* color space and 16-bit encoding to work without missing information. The developed solution exploits the RAW decoder DCRaw, setting a 16-bit output with fixed white level, gamma equal to 1 and ignoring the image own histogram. This allows to perform both linearization and conversion of linearized values into CIE*XYZ* tristimulus values. It is a well-known fact that a channel response processing by on-board software is introduced by manufacturers to minimize CCD construction problems, but this handling generates non-linearities. However, we do not introduce further processing to fix these non-linearities than the modifications already made by DCRaw. Although this solution is not accurate as the ‘ad hoc’ solution illustrated in [38], we ensure that the final result is sufficiently accurate due to the DCRaw ability to take into account the camera's built-in features.
(c) *Exposure equalization and white balance.* First step of color correction consists of a white balance and exposure equalization against a specific patch. White balance was performed with a linear transformation against the patch D4 of the CC. As demonstrated in Viggiano [77] white balancing using an illuminant-dependent characterization produce consistently good results.
(d) *Denoise.* To limit image noise and their amplification in color correction a solution for evaluating noise before and after color correction and automatically applying the denoise algorithm to the corrected image has been developed. The solution suggested here is based on three observations:
  - noise can be very low in digital SLRs, and practically reduced to zero for low speed;
  - as the camera ISO increase, the amount of noise automatically increases as well;
  - data captured from the color filter array (CFA) cannot be displayed before further steps: white balance and demosaicing and the color transformations, which adapt the linear to the lightness data to those adapted to the monitor gamma and color space. Through demosaicing the noise is spatially and chromatically correlated and through the nonlinear color transformations the noise distribution is unknown. As this last noise characteristic cannot be easily incorporated into the denoising methods, denoising has been applied to the raw CFA data (the mosaicked data linear to the light intensity with uncorrupted noise characteristics) exploiting the observation of [78] that showed as in the raw data noise presents a known distribution, and a signal-dependent variance, which can be precisely estimated based on measurements.

The implemented solution is a variant of the state of the art method in image denoising, named Block Matching 3D (BM3D) [57], combined with a rough but efficient noise estimation algorithm. The developed technique (so-called *CBM3D-new*) is created starting from the CBM3D method [79], an extension of the multi-stage approach of BM3D via the YoUoVo color system. CBM3D yields a basic estimate of the image, using the luminance data. The denoised image is carried out performing a second stage on the color channels separately. *CBM3D-new* automates the CBM3D processing selecting the necessary parameters to initialize the algorithms using EXIF data (type of camera and ISO sensitivity) and a series of stored profiles obtained from experimental trials [9].

(e) *Polynomial fitting*. The generic formula of the polynomial model is as follows:
  (6) $$X_{k}=\underset{0\leq j_{1}+j_{2}+j_{3}\leq n}{\sum }m_{X,j_{1},j_{2},j_{3}\;}R_{k}^{j_{1}}G_{k}^{j_{2}}B_{k}^{j_{3}}\;Y_{k}=\underset{0\leq j_{1}+j_{2}+j_{3}\leq n}{\sum }m_{Y,j_{1},j_{2},j_{3}\;}R_{k}^{j_{1}}G_{k}^{j_{2}}B_{k}^{j_{3}}\;Z_{k}=\underset{0\leq j_{1}+j_{2}+j_{3}\leq n}{\sum }m_{Z,j_{1},j_{2},j_{3}\;}R_{k}^{j_{1}}G_{k}^{j_{2}}B_{k}^{j_{3}}\;k=1,\;2,\;3,\;\ldots ,\;k$$
  where *X_k_*, *Y_k_* and *Z_k_* are the CIE tristimulus values of the *k*^th^ sample, *R_k_*, *G_k_* and *B_k_* are camera signals of the *k*^th^ sample, *K* is the number of samples in the characterization target, *n* is the order of the polynomial, *j_1_, j_2_* and *j_3_* are non-negative integer indices and *m_X_*, *j_1_, j_2_*, *j_3_*; *m_Y_*, *j_1_, j_2_*, *j_3_*; and *m_Z_*, *j_1_, j_2_*, *j_3_*; are the model coefficients to be determined. Equation (6) can be expressed in matrix form:
  (7) $$g_{k}=Mf_{k}$$
  where *f_k_* is the *k*^th^ camera response vector formed by the given *RGB* vector, *g_k_* is the *k*^th^ tristimulus value vector obtained by physical measurement using a spectrophotometer and *M* is the mapping matrix to transform *f_k_* to the vector *g_k_* of tristimulus values. Also for the polynomial regression a least-squares method can be used to determine the matrix *M* based on the known camera response vector *f_k_* and the corresponding measured tristimulus values *f_k_*. Letting:
  (8) $$G=\left[g_{1},\;g_{2},\;\ldots ,\;g_{k}\right]\;\mathrm{and}\;F=\left[f_{1},\;f_{2},\;\ldots ,\;f_{k}\right]$$
  results in a matrix equation:
  (9) G=MF
  where *M* is a 3 by *N* matrix, *F* is an *N* by *K* matrix and *G* is a 3 by *K* matrix; *N* is dependent on the order *n*. In the above matrix equation, the matrices *G* and *F* are known and *M* is unknown. The least-squares solution can be modelled as:
  (10) $$Minimize:{\left\|MF-G\right\|}_{2}$$
  where ${\left\|MF-G\right\|}_{2}$ is the two-norm, i.e., the mathematical measure of length given by ‘the square root of the squares’. When *K* = *N*, the problem in Equation (10) has a unique solution:
  (11) $$M=GF^{-1}$$
  where *F^−1^* is the generalized inverse of matrix *F*. It is noted that the size of the column vector *f_k_* is determined by the order n of the polynomial according to Equation (7).

The solution used in our case is a per-channel weighted polynomial curve fitting algorithm: the MATLAB function *Weigthed Polyfit (x,y,n)*. The function accepts as input the normalized color matrix of the 24 CIE*XYZ* values on the patch of the target image, and the normalized destination color space (in this case sRGB) measured values of the patches. It returns a structure containing the parameters needed to best fit (in a least-squares sense) by relating the values of the patches of the current image (the observed data) (*y*), with the measured reference values (the expected data) (*x*). Goodness of fit coefficient and polynomial degree are selected using a weighted fit minimizing the RMSE:

(12) $$RMSE=\sqrt{\overset{n}{\underset{i=1}{\sum }}\omega_{i}{\left(\hat{x_{i}}-x_{i}\right)}^{2}}$$

where ω is the weight of each patch of the CC. After some experiments using equal weighting and the trendy weight according to CIELAB L*, giving more weight to visually-prominent highlight patches, the weight of the greyscale row patches was selected as double of the weight of the colored ones. This is driven by three different motivations:

a) Polynomial regression usually minimizes only the average color error, not guaranteeing a uniform accuracy across the entire color range. We observed that the preservation of achromatic colors as achromatic allows to minimize peak of error and to minimize white point and exposure errors, i.e., necessary prerequisites for hue-plane preservation;
b) The weighted least squares solution assumes response data of equal quality and, therefore, with constant variance. In presence of poor quality data (a condition not unusual in the field), this assumption is violated and the fit might be incorrect.
c) A higher color dominant in the corrected image (even with a low mean ΔE*_00_ error) does not allow good results with the ACR scripts algorithm, which tends to converge towards less efficient solutions than the ones achieved with images where the achromatic component is correct.

The doubled weight is the result of experimental analysis, in which a numerical value equal to 1, 2, 4, 8 was applied each time as a weight on a set of 30 selected target images, having different characteristics.

(f) *Color correction refinement.* This step consists on a rewritten MATLAB version of the Adobe Camera Raw (ACR) calibration scripts coming from Bruce Fraser’s calibration procedure for successive approximations [80]. This algorithm allows to optimize polynomial regression results and to avoid error due to illumination changes. It exploits a feature on which ACR is originally based to overcome the problem that most cameras respond very differently to tungsten light than to daylight because of their much more sensitivity to red light than to blue. For each supported camera, Camera Raw contains two built-in profiles: one related to a tungsten light source, one related to a daylight light source. Camera Raw’s White Balance tools—the Temperature and Tint sliders—interpolate between (or even extrapolate beyond) these two built-in profiles.
(g) *Color space choice and gamma.* In the color correction process, a key-point is the use of appropriate color spaces in which to apply the color correction algorithms given above and the scene-referred non-linear color space (e.g., sRGB, AdobeRGB (1998), DCI-P3) for the output images [81]. The processing color space used is the CIEXYZ taking in account the consideration that using this color space, errors calculated by the least squares fitting algorithms allows to produce final corrected images that look more closely matched to the original image [82]. Our final color space is the rendered space sRGB, the today IEC 61966-2-1 default color space for multimedia application [83]. The standard defines the relationship between the 8 bit sRGB values and the CIEXYZ values measured in comparison to the reference CRT monitor. The ITU-R BT.709 define the reference monitor’s white-point and colors, at 6500 K temperature (D65). The sRGB IEC 61966-2-1 is an excellent solution, because it is consistent from color capture with different acquisition devices (scanner and cameras), to visualization by different monitors or video-projectors. The two dominant programming interfaces for 3D graphics, OpenGL [84] and Microsoft Direct3D [85], have both incorporated support for the sRGB color space. Main downside of the sRGB color space is the gamma value built inside: the sRGB gamma cannot be expressed as a single numerical value. The overall gamma is approximately 2.2, but it consists of a linear (gamma 1.0) section near black, and a non-linear section elsewhere comprising a 2.4 exponent and a gamma changing from 1.0 through about 2.3. A second drawback concerns colors gamut, narrower than the human one. In detail sRGB color gamut does not allow to display properly saturated colors such as yellow cadmium and blue cobalt. This last weakness is not a problem in our case studies, because misrepresented colors are rarely found.

Another important consideration is the use of gamma corrected color spaces. While ‘Gamma Correction’ was originally intended to compensate the non-linear response of the CRT monitor, by a remarkable coincidence the non-linear response of the human visual system is approximately the inverse of the nonlinear response of a CRT monitor. Thus, gamma pre-corrected color signals are already approximately perceptually coded. Technically speaking in the ‘Gamma Correction’ model, the input is linear brightness, with values from 0 (black) to 1 (full brightness) and is applied over each *RGB* color component. The output is the ‘Gamma Corrected’ (non-linear) brightness. The ‘Gamma Correction’ function has the form *y = x^γ^*, where the exponent is a numerical constant known as gamma. In the case of digital cameras, the per channel ‘Gamma Correction’ function is as follows:

(13) $$new\_val={\left(\frac{old\_val}{max\_val}\right)}^{y}\cdot max\_val$$

where *max_val* is the maximum allowed value (i.e., 255 for 8 bit-depth representation).

In our workflow, before color balance, considering that the linear brightness is not perceptually linear, a ‘Gamma Correction’ is applied. The value *γ* = 2.2 is used, well-fitting the behavior of the output color space sRGB. This operation converts all images into the camera’s native linear color space, refining their quality.

## 5. Performance Evaluation

**Figure 5:**
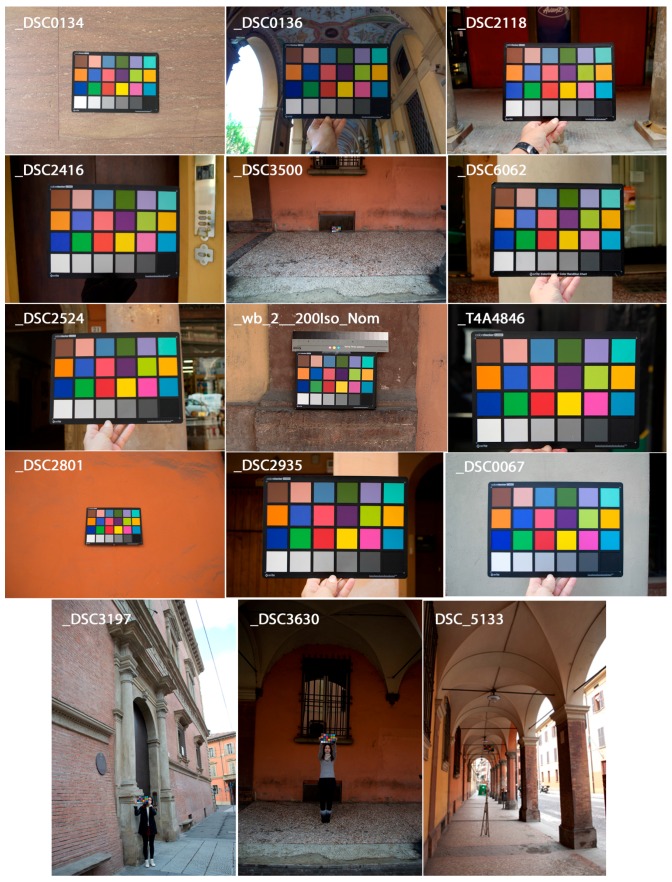
Dataset of 15 RAW images with CC evaluated.

**Figure 6:**
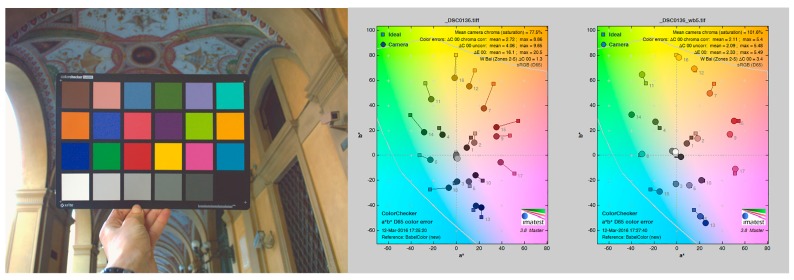
Mean camera chroma relative to the mean ideal chroma in the CIE color metric and the color analysis for the left image: manual color balance (center) and automated workflow results (right).

For the evaluation of the performances of the implemented methodology, different functionalities were tested in the context of architectural scenarios:

A) *ColorChecker finding*: a standard dataset of RAW camera images (i.e., free of any color correction) in which a known color target was used (Figure 5). Three cameras (Nikon D200, Nikon D3100 and Nikon D5300 (www.nital.it)) with wide-angle lenses were used in the acquisition procedure. The dataset consisted of 15 images representing different cases and problems and the CC chart was included in every acquisition. The presented method is compared to [70] and X-Rite ColorChecker Passport Camera Calibration Software [65] on the set of 15 images depicting the target. Results are shown in Table 1, demonstrating that the presented solution outperforms the other two detection algorithms.
B) *Color fidelity*: the evaluation of color accuracy, based on a CC target, comprises: a physical reference chart acquired under the current illumination (corresponding to the illumination to be discard); a reference chart color space with the ideal data values for the chart; a way to relate or convert the device color space to the reference chart color space; and a way to measure and show errors in the device’s rendering of the reference chart. Color accuracy was computed in terms of the mean camera chroma relative to the mean ideal chroma in the CIE color metric (ΔE*_00_) as defined in 2000 by the CIE on the CIEXYZ chromaticity diagram (Figure 6). The ΔE*_00_ calculation was made using the ColorChecker tool of Imatest Master software version 4.5 [66]. Exposure error in f-stops was also evaluated. Best results are obtained if it is less than 0.25 f-stops. The ΔE*_00_ of the automated pipeline was also compared to the result of the previous manual workflow for a subset of targets used in *A.* Results are in Table 2 where a great improvement over the manual solution is evident.

The extra-step consisting in the ACR scripts-based refinement performs well, ensuring a further improvement for cases where the polynomial techniques have limitations (i.e., targets in images _T4A4846, _wb_2__200 presenting little exposure differences across the target).

**Figure 7:**
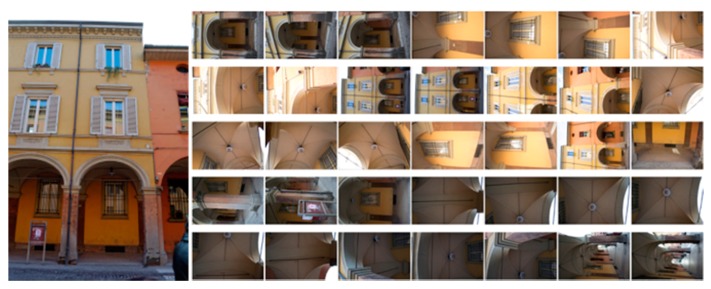
The building with portico dataset.

**Figure 8:**
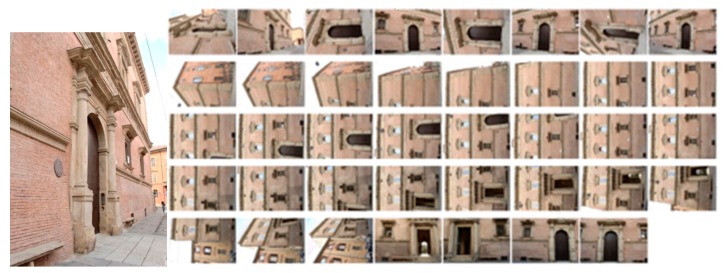
The Palazzo Albergati dataset.

C) *Photogrammetric pipeline*: the performances of the previously presented pre-processing are evaluated in the photogrammetric pipeline and reported using the pairwise matching efficiency, the statistical output of the bundle adjustment (re-projection error) and the number of points in the dense point cloud. Image processing has been performed using a combination of a calibrated SIFT version as detector/descriptor [86], VisualSfM as bundle adjustment [87] and n-frames SURE [88] for dense image matching. The scaling is given by topographic network. Two different image networks with different imaging configurations, textureless areas and repeated pattern/features were employed. The two datasets represent an urban test framework and summarizes a typical historical urban scenario (Figure 7 and Figure 8). Both datasets were acquired using a Nikon D3100 with an 18-mm nominal focal length and consist of convergent images and some orthogonal camera rolls. They feature many situations emblematic of failure cases, i.e., 3D scenes (non-coplanar) with homogeneous regions, distinctive edge boundaries (i.e., windows, doors, cornices), repeated patterns (recurrent architectural elements, bricks, etc.), textureless surfaces and illumination changes.

The first dataset (building with portico/35 images) pertains two spans of a three-storey building (6 × 11 m) characterized by arches, pillars/columns, cross vaults and plastered walls (Figure 7). Camera moved along the porticoes, with some closer shots of the columns. The second dataset (Palazzo Albergati/39 images) depict a three-storey historical palace (54 × 19 m) characterized by repeated brick walls, stone cornices and a flat façade (Figure 8). Camera moved along the façade of the building, with some closer shots of the entrances.

**Figure 9:**
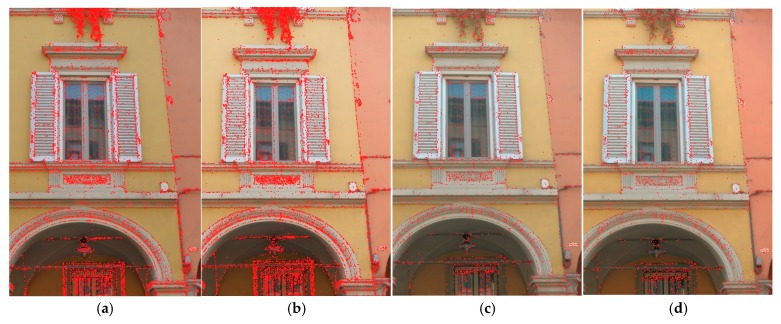
Results of SIFT interest point detection using different color enhancements: (**a**) no enhancement; (**b**) manually enhanced; (**c**) automatically enhanced; (**d**) automatically enhanced using the whole pipeline (polynomial regression + ACR based correction). Although in (**d**) the number of detected points is lower, they are more robust and reliable, allowing better orientation procedures.

**Figure 10:**
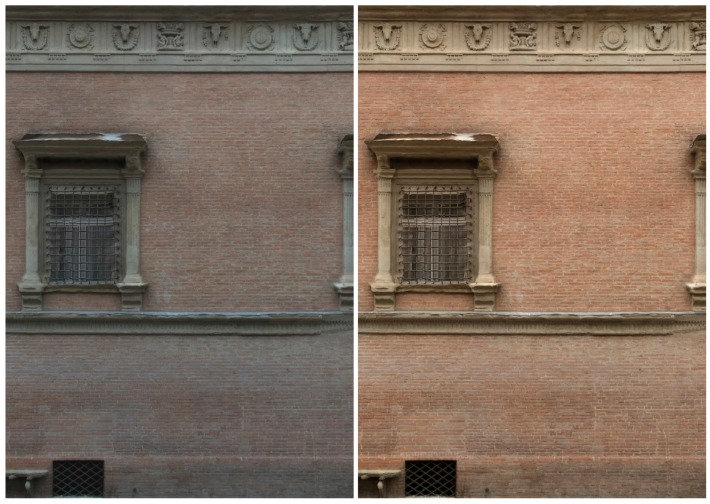
Bologna, Palazzo Albergati: comparison between the textured 3D model without (**left**) and with color correction (**right**).

**Figure 11:**
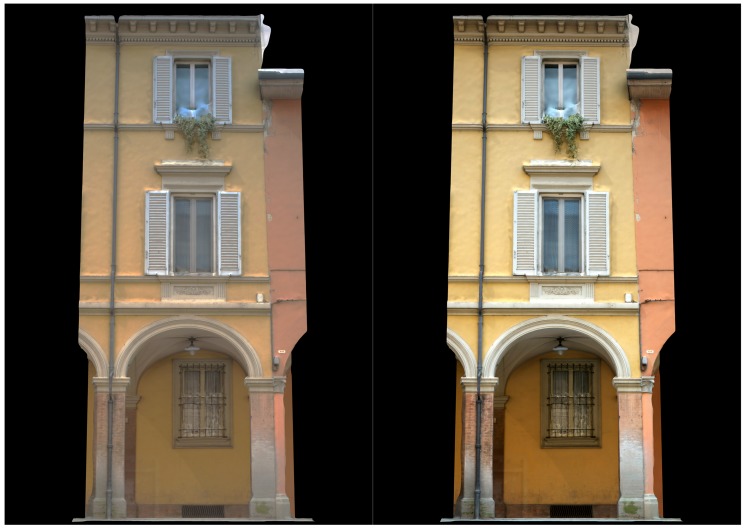
Bologna, building with portico: comparison between the textured 3D model without (**left**) and with color correction (**right**).

Figure 9 shows the typical effects of the enhanced pipeline on SIFT points detection. The number of points detected is lower but these points are more robust and reliable, allowing to avoid some well-known SIFT failures. Table 3 and Table 4 report the results of the performed tests, showing an excellent improvement with the automation both in the number of images oriented and in the dense stereo matching phase. Figure 10 and Figure 11 compare 3D textured models without (left) and with the developed automated color correction (right).

## 6. Conclusions and Recommendations

The area of faithful color reproduction in 3D modeling application is still of great interest for the research community. Indeed, even in commercial software, color management is normally limited to simply lightness adjustment and this generates significant artifacts and color discrepancies on the final 3D model. This is a major issue especially in areas like Cultural Heritage where faithful color reproduction is a key requirement.

In this paper, a new automatic solution to: (i) ensure color fidelity in automatic photogrammetry and (ii) increase the processing performances of the photogrammetric pipeline was presented. The method exploits color balance by target-based polynomial techniques.

The development comes from manual or semi-automatic solutions that we have been experiencing for years, whose efficiency is known and codified. Now these solutions were automated and some well-known limitations overcome, both in terms of quality of the results and dependence on expensive commercial software (e.g., Adobe Photoshop). The developed solution could be directly and immediately applied in any photogrammetric pipeline, in order to appreciably increase the accuracy of the color characterization of the cameras, without any need to change the rest of the workflow. The solution automatically carries out five tasks:

- Target detection.
- Exposure equalization and white balance.
- Establishment of relationship between the spectral responsivities of the camera and the CIE color matching function.
- Color balance of the image.
- Color space assignment (i.e., sRGB color space).

The developed technique well performs in all pipeline phases of target recognition, color calibration and photogrammetric processing of typical architectural heritage scenarios. The presented method outperformed two popular algorithms in automatic ColorChecker finding. The presented results demonstrate a stable and widely applicable color fidelity/accuracy outcome, based on (i) a CC target identification; (ii) a specific polynomial function and (iii) a MATLAB implementation of the ACR scripts to refine the results ensuring further improvement. Moreover, the presented method allows to improve the whole photogrammetric pipeline, from tie point identification to bundle adjustment and dense image matching phase.

**Figure 12:**
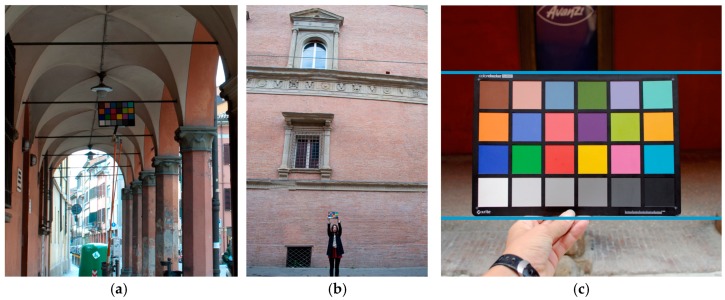
Some issues related to photographing the CC in order to avoid any affect to the quality of calibration: (**a**) ambient light variations causing changes in the color values and high color; (**b**) a small size of chart in the image; (**c**) a geometric deformation of shape of target (i.e., orientation and location of chart in the image, not rigid material of color chart and camera optics, etc.).

As the image pre-processing is completely automated, the preservation of color fidelity needs some practical recommendations mainly concerning the CC image capture phase:

- taking pictures in the most possible homogeneous operative conditions (aperture/exposure direction and intensity of light);
- taking pictures with overcast sky, so you have a diffused and uniform lighting with no obvious dominant color of the sunlight, and without shadows projection;
- keeping CC surfaces with an angle of incidence with sunlight around 20–45° or less;
- placing the CC, if possible, on a tripod, orthogonal to the camera optical axis to minimize the light glare and with a dark background to facilitate its detection;
- taking care to avoid saturating any of the patches;
- taking CC image width of 500 to 1500 pixels to ensure a correct ΔE_00_ analysis.
- Other issues related to CC images affecting the quality of calibration could be (Figure 12):
- the position of the target far from the photo center point;
- the rough and irregular nature of the subject that could prevents to have CC always illuminated with the same angle of incidence, thus defeating their performance monitoring tools;
- less reliable color balance results as wider is the angle between camera axis and the plane where the target lays and/or the difference between its light reflectance index and artifact one. Cause these conditions the CC leans to reflect more or less light than the material where is placed;
- the distance from the camera to the CC is not critical. There is no need to fill the frame with the CC image, especially for high resolution cameras. Filling the frame may reduce accuracy if there is significant vignetting;
- problems related to the position of the target in the image are minimized by the new automatic detection technique able to find CC of different size and in different position.

## Figures and Tables

**Table 1:** Number of ColorCheckers detected by different algorithm.

	Our Solution	Enhanced CCFind	X-Rite ColorChecker Passport Camera Calibration Software
Number of ColorCheckers detected	15	13	7

**Table 2:** ΔE*_00_ evaluation.

File Name	ΔE*_00_ Manual sRGB	ΔE*_00_ Automated sRGB	ΔE*_00_ Automated ACR sRGB	Exposure Error (f-Stops) Manual sRGB	Exposure Error (f-Stops) Automated sRGB	Exposure Error (f-Stops) Automated ACR sRGB
_wb_2__200	4.65	2.08	1.86	0.08	0.01	0.03
_DSC2524	3.84	1.56	1.56	0.13	0.02	0.02
_T4A4846	4.42	4.19	4.09	0.07	−0.02	0.06
_DSC2801	4.36	1.85	1.59	0.18	0.02	0.09
_DSC2935	3.92	2.83	2.79	0.09	0.04	0.18
_DSC0134	4.47	2.29	2.29	0.19	0.01	0.01
_DSC0136	16.1	2.85	2.85	1.19	0.00	0.00
_DSC2118	7.98	1.75	1.75	0.41	0.02	0.02
_DSC2416	5.92	2.60	2.60	0.09	0.02	0.02
_DSC6062	5.71	1.87	1.73	0.04	−0.02	−0.04
**Mean error**	**6.137**	**2.387**	**2.311**	**0.247**	**0.010**	**0.044**
**Standard deviation**	**3.714**	**0.779**	**0.796**	**0.348**	**0.019**	**0.060**

**Table 3:** “Palazzo Albergati” dataset—photogrammetric process results.

	No Enhancement	Manually Enhanced	Automatically Enhanced	Automatically Enhanced—ACR
Oriented images	34	39	39	39
RMS re-projection error	0.286	0.185	0.313	0.250
Points from more than 3 cameras	51,163	197,969	82,988	115,048
3D points in the dense point cloud	65,194,066	80,369,256	80,473,354	80,731,021
Inlier matches _DSC7061 _DSC7062	7770	6095	6028	6131

**Table 4:** “Building with portico” dataset—photogrammetric process results.

	No Enhancement	Manually Enhanced	Automatically Enhanced	Automatically Enhanced—ACR
Oriented images	26	27	28	32
RMS reprojection error	0.816	1.050	0.558	0.536
Points from more than 3 cameras	6005	7967	7645	7729
3D points in the dense point cloud	21,116,172	23,807,743	19,165,033	23,348,061
Inlier matches_DSC6702 _DSC6703	1985	967	2082	2001
